# Masked inv dup(22)(q11.23), tetrasomy 8 and trisomy 19 in a blast crisis-chronic myeloid leukemia after interrupted Imatinib-treatment

**DOI:** 10.1186/s13039-015-0204-x

**Published:** 2015-12-23

**Authors:** Abdulsamad Wafa, Suher Almedani, Thomas Liehr, Walid Al-Achkar

**Affiliations:** Department of Molecular Biology and Biotechnology, Atomic Energy Commission, Human Genetics Division, P.O. Box 6091, Damascus, Syria; Jena University Hospital, Institute of Human Genetics, Jena, Germany

**Keywords:** Chronic myeloid leukemia, Philadelphia chromosome, inv dup(22)(q11.23), Tetrasomoy 8, Clonal evolution, Prognostic factors

## Abstract

**Background:**

The Philadelphia (Ph) chromosome, or derivative chromosome 22 [der(22)], is a product of the reciprocal translocation t(9;22). It is the hallmark of chronic myelogenous leukemia (CML). It results in juxtaposition of the 5’ part of the *BCR* gene on chromosome 22 to the 3’ part of the *ABL1* gene on chromosome 9. During CML progression 60–80 % of the cases acquire additional genetic changes. Blast crisis (BC) is characterized by the rapid expansion of a population of differentiation arrested blast cells (myeloid or lymphoid cells population), often presenting with secondary chromosomal abnormalities. Here we report an unusual CML-BC case with acquired secondary chromosomal aberrations observed after the patient had to interrupt a successful Imatinib treatment for overall 16 months.

**Case presentation:**

A complete cytogenetic and molecular cytogenetic analysis were performed and application of molecular genetic methods such as reverse transcription polymerase chain reaction (RT-PCR) finally characterized a complex karyotype including an inv dup(22)(q11.23), tetrasomy 8 and trisomy 19.

**Conclusions:**

Here we report the first case of a BC after successfully initiated and suddenly interrupted Imatinib treatment. Changes present after such an instant indicate for a rapid progression after Imatinib is no longer suppressing the disease.

## Background

Chronic myeloid leukemia (CML) is a myeloproliferative disorder associated with the presence of Philadelphia (Ph) chromosome or also is called a derivative chromosome 22 [der(22)]. The latter is found as sole aberration in more than 90 % of the early chronic phase (CP) CML cases and it is due to a reciprocal translocation t(9;22)(q34.1;q11.2). This rearrangement involves the breakpoint cluster region (*BCR*) gene 22q11.2 and the c-Abelson proto-oncogene (*ABL1*) gene in 9q34, generating a chimeric BCR-ABL gene that encodes a constitutively activated protein tyrosine kinase. The kinase activity of BCR-ABL is essential for the pathogenesis of CML [1].

Additional genetic changes occur in less than 10 % of CML cases at diagnosis; other genetic changes are detectable in 60-80 % of the cases in advanced disease [accelerated phase (AP) and blast crisis (BC)] [1]. The correct identification of such abnormalities is important for diagnosis of the diseases determined by the WHO Tumor Classification [2] and for treatment purposes. The first therapeutic choice, tyrosine kinase inhibitors, has shown great therapeutic efficacy [3].

Imatinib mesylate (IM = Glivec, formerly called STI571) is a chemically designed drug able to block BCR/ABL1 tyrosine kinase activity and is successfully used as a first-line therapy for all CML patients [4]. It has significantly improved the prognosis especially for newly diagnosed CML-CP patients, in which the complete hematologic response rate was more than 90 %, and the complete cytogenetic response rate 70-80 %. However, IM resistance has emerged as an important clinical challenge. Multiple factors may contribute to IM resistance including mutations of BCR-ABL1 kinase domain, BCR-ABL1 amplification or enhanced expression rates, clonal evolution, and decrease in imatinib bioavailability or cell exposure [5]. Amplification can be determined by fluorescence in situ hybridization (FISH) or comparative genome hybridization (CGH), while other aforementioned developments are difficult to monitor.

Here we presented a new CML-BC case with complex karyotypic changes including an inv dup(22)(q11.23) in a patient with an interrupted IM treatment; to the best of our knowledge the outcome of such a case was not previously reported.

## Case presentation

In June 2011 a 62-year-old Syrian male was diagnosed as suffering from CML. Physical examination revealed splenomegaly, which was the indicative symptom. Routine peripheral blood test showed elevated white blood cells (WBC) of 170.1 × 10^9^/l (43.2 % neutrophils, 7.9 % lymphocytes, 11.2 % monocytes, 28.1 basophiles, and 9.6 % eosinophiles), red blood cell (RBC) count was 3.05 × 10^6^/mm^3^, hemoglobin level was 6.1 g/dl and the platelet count was 1,039 × 10^9^/l. The patient was diagnosed as CML-AP according to WHO recommendations. The patient did not receive any previous treatment.

The patient was referred for a second time in July 2012 and was successfully treated with IM (400 mg/day) for 13 months. The more recent routine peripheral blood test was: WBC 5.3 × 10^9^/l (52.1 % neutrophils, 34.8 % lymphocytes, 10.5 % monocytes, 0.5 basophiles, and 2.1 % eosinophiles), RBC count was 2.98 × 10^6^/mm^3^. The platelet count was 203 × 10^9^/l and the hemoglobin level was 10.9 g/dl. Serum lactate dehydrogenase value (LDH) was 512 U/l (normal level <460 U/l).

The patient was referred for a third time in May 2014 after he stopped IM treatment for 16 months due to political situation in his home country. WBC was then 1,334.0 × 10^9^/l (92 % of cells were blasts), red blood cell (RBC) count was 1.91 × 10^6^/mm^3^, hemoglobin level was 6.1 g/dl and the platelet count was 56 × 10^9^/l. The patient was diagnosed as CML-BC according to WHO recommendations, in a high Sokal risk of 2,594 (0.8–1.2), high Hasford (Euro) risk of 6039 (>1,480), and Etous probability of no complete cytogenetic response (CCgR) at 18 months was 11 %. Later the patient was lost during follow-up.

## Results

**Fig. 1:**
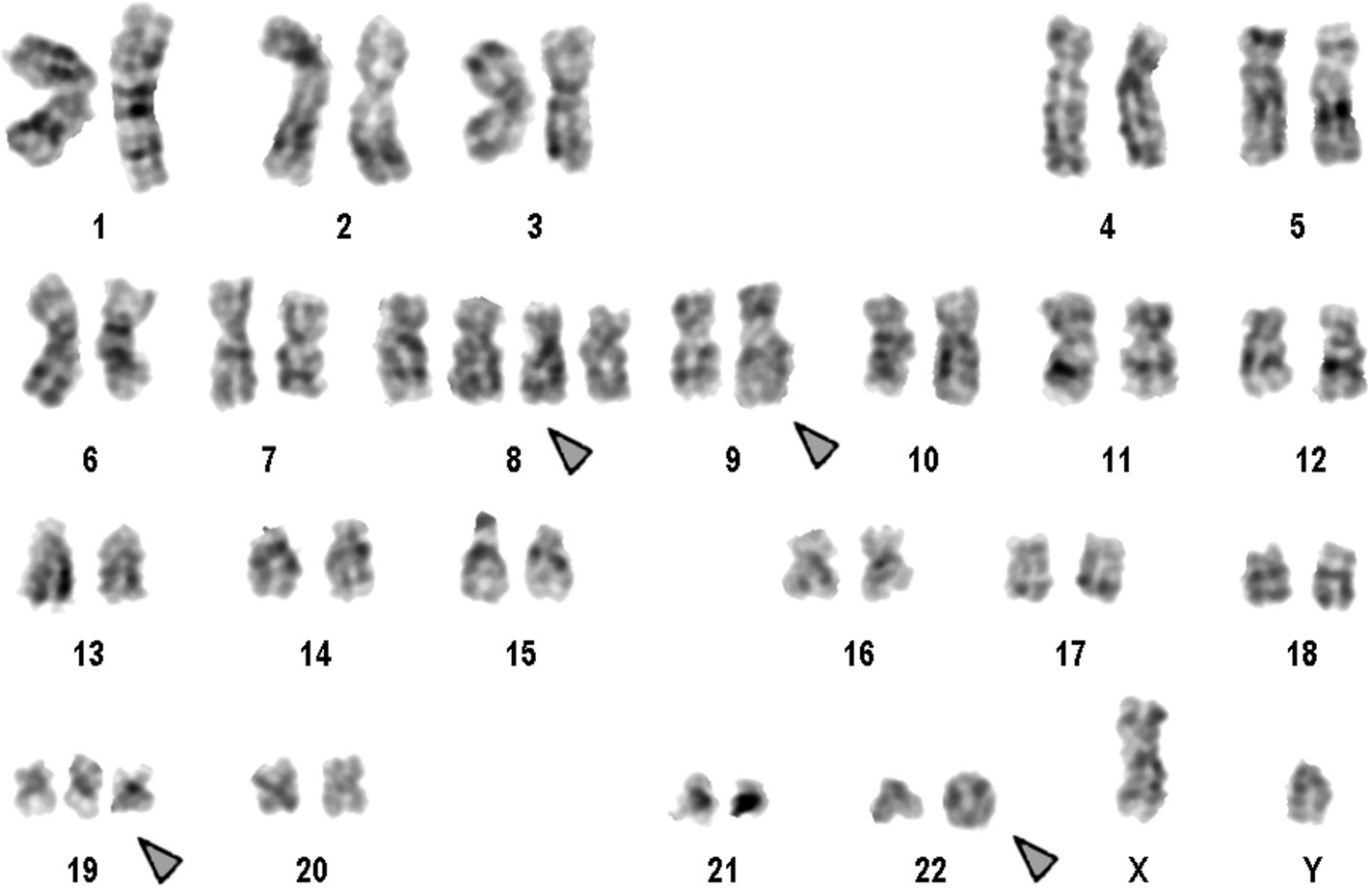
GTG-banding revealed a 49,XY,+8,+8,t(9;22),+19,inv dup(22). All derivative chromosomes are shown and with arrow

**Fig. 2:**
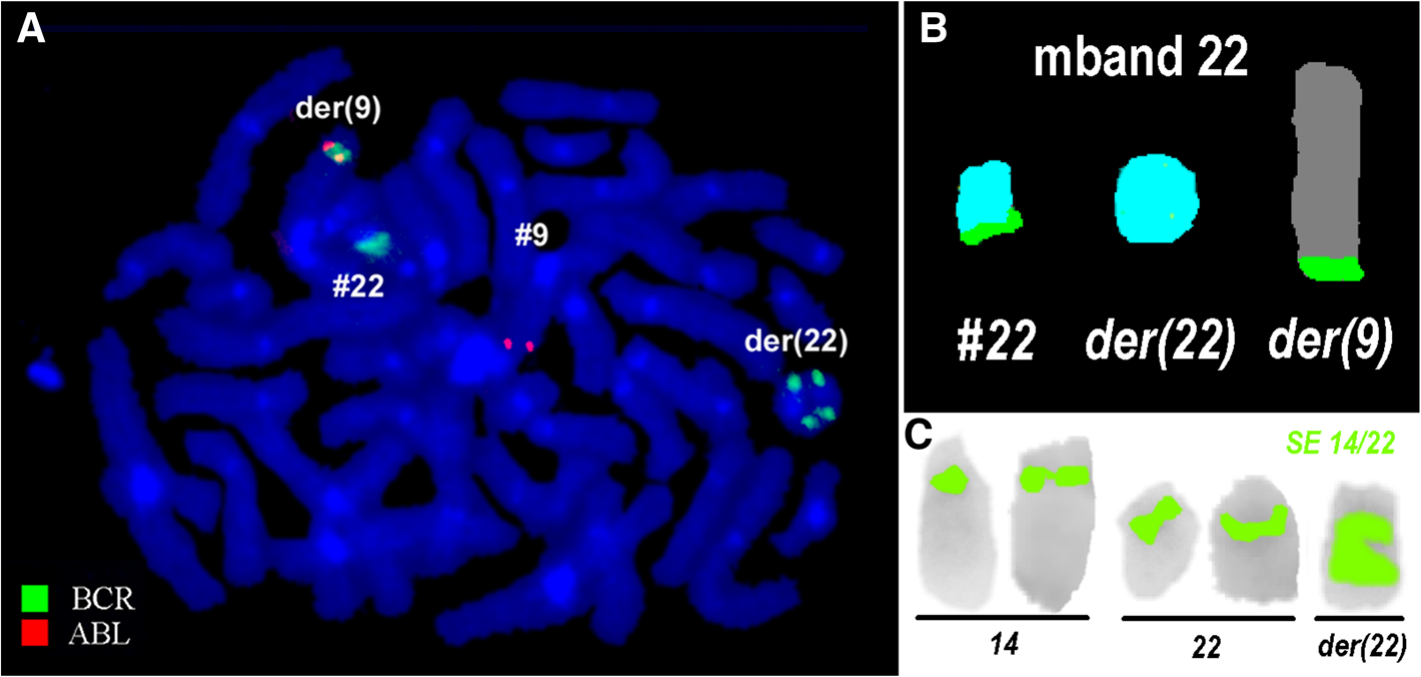
Karyotype and chromosomal aberrations were confirm ed using molecular cytogenetic approaches. **a** FISH using probes for BCR (green) and ABL (red) showed one red signal on normal chromosome 9; three green signals, one on normal chromosome 22 and two on der(22); BCR/ABL1 fusion on der(9) with absent of an *ABL1* signal on der(22). **b** The application of MCB (22) characterized the inv dup(22)(q11.23) comprehensively. **c** FISH using alpha satellite probe for chromosomes 14 and 22 together revealed two centromers on der(22). Abbreviations: # = chromosome; der = derivative chromosome

Prior to the IM-treatment banding cytogenetics revealed a karyotype of 46,XY,t(9;22)(q34.1;q11.2)[20]. After 13 month IM-treatment the karyotype was 46,XY,t(9;22)(q34.1;q11.2)[11]/46,XY[9]. However, after interrupted involuntarily IM-treatment for 16 months banding cytogenetics revealed a karyotype of 49,XY,+8,+8,t(9;22)(q34.1;q11.2),+19,inv dup(22)[6]/48,XY,+8,+8,t(9;22)(q34.1;q11.2),inv dup(22)[1]/48,XY,+8,t(9;22)(q34.1;q11.2),+19,inv dup(22)[3]/46,XY[10] (Fig. 1). Further on molecular cytogenetic studies were performed (Fig. 2). Dual-color-FISH prior and post to IM-treatment using a specific probe for BCR and ABL1 revealed two fusion signals, on the der(9) and der(22), respectively (data not shown). Whereas, after interrupted IM-treatment there were: one BCR/ABL1 fusion signal on a der(9) one red signal on chromosome 9, and three green signals, one on chromosome 22 and two on an inv dup(22) (Fig. 2a). Chromosomes 9 and 22 were studied with whole chromosome painting (WCP) probes and did not provide any hint on cryptic translocations (data not shown). Multicolor banding (MCB) using a specific probe for chromosome 22 revealed a inversion and duplication of der(22) (Fig. 2b). A probe specific for alpha satellite regions of chromosomes 14/22 probe confirmed the presence of an inv dup(22) (Fig. 2c). RT-PCR prior and after the IM-treatment confirmed the presence of the BCR-ABL1 fusion (b3a2 transcript) revealing a major M-BCR transcript, most often identified in CML (data not shown). Thus, the following final karyotype after interrupted IM-treatment was determined:

**Table:** 

49,XY,+8,+8,t(9;22)(q34;q11.2),+19,inv	dup(22)(::q11.23- > p13::p13-
>q11.23:)[6]/48,XY,+8,+8,t(9;22)(q34;q11.2),inv	dup(22)(:q11.23- > p13::p13-
>q11.23:)[1]/48,XY,+8,t(9;22)(q34;q11.2),+19,inv	dup(22)(:q11.23- > p13::p13-
>q11.23:)[3]/46,XY[10].	

## Conclusions

According to the literature, BC of CML may be myeloid or lymphoid. Approximately 15–20 % of patients in BC retain the Ph + cell population unaltered, whereas 80–85 % of the cases undego karyotypic evolution [1]. The most common secondary chromosomal aberrations in addition to a Ph-chromosome are +8, +Ph, i(17q) and +19. Other chromosomal aberrations are less frequent, as -Y, +21, +17, −7, and −17 [1, 6]. Hyperdiploidy is not common in CML cases [7], however it is a common finding in advanced phase-CML patients [6, 8], and it was already reported in one CML-CP patient as a secondary chromosomal aberration after IM therapy [9].

The der(22) inv dup(22)(q11.2)t(9.22)(q34;q11.2) is an uncommon event in CML-advanced phase. However, this chromosomal aberration was observed in 6 cases before (the majority were CML-advanced phase), and it was product of a der(22)t(9;22)(q34;q11.2); the BCR/ABL1 fusion remained on such inv dup(22) derivatives [10-13]. In the present case, we noted an inv dup(22)(q11.23) without presence *ABL1* signal on derivative 22. To our knowledge, BC characterized by a inv dup(22)(q11.23) without presence *ABL1* signal on inv dup(22) associated with tetrasomy 8 and trisomy 19 has not been reported together in CML, yet [14]. Maybe submicroscopic structures of DNA-sequences in chromosome 22 are herfore responsible [15] However, tetrasomy 8 is not a rare event in advanced phase-CML; more than 50 such cases are listed in Mitelman Database [14].

Clinical resistance by BCR-ABL1 amplification mechanism is uncommon, but not a really rare event in CML resistance to treatment. Amplification of the Ph chromosome on conventional karyotyping may present as double Ph, dicentric Ph, double minutes, or masked Ph and inverted duplication of Ph [13, 16]. However, inverted duplication is unique structure and it is related to a DNA amplification event, which led to increased expression of bcr-abl protein [17, 18]. Thus, the inv dup(22) derivative is not easily connected with IM-resistance.

CML patients in AP and myeloid BC treated with 400 or 600 mg of IM can show major cytogenetic response [9, 10, 19]. However, our patient achieved only a minor cytogenetic response followed 13 months of IM treatment. Aberrations like inv dup(22)(q11.23) without presence *ABL1* signal on inv dup(22) associated with tetrasomy 8 and trisomy 19 were noted after the reported patient stopped IM treatment involuntarily for overall 16 months. Thus, it is unclear, still unlikely, that such kind of karyotypic changes might have been seen in this patient in connection with IM-resistance development. In conclusion the present case is one of the rare examples, where infortune political circumstances lead to unintentional interruption of a successful IM-treatment in a Syrian CML-patient. It is thus a unique example for which cytogenetic changes may appear during 16 month interruption of IM-treatment. Trisomy 8 and 19 have earlier been reported as secondary events in untreated or treated CML in BC. The inv dup(22)(q11.23) seen here may be a secondary finding appearing after interrupted IM-treatment.

## Materials and Methods

### Chromosome analysis

Chromosome analysis applying GTG-banding according to standard procedures [20] was performed prior IM treatment. 20 metaphase cells derived from unstimulated bone marrow culture were analyzed. Karyotypes were described according to the International System for Human Cytogenetic Nomenclature (ISCN 2013) [21].

### Molecular cytogenetics

Fluorescence in situ hybridization (FISH) using the LSI BCR/ABL dual color dual fusion translocation probe (Abbott Molecular/Vysis, Des Plaines, IL, USA), alpha satellite probes for chromosomes 14 and 22 (Qbiogene, MP Biomedicales, Santa Ana, CA, USA) were applied together with whole chromosome painting (WCP) probe for chromosomes 9 and 22 (MetaSystems, Altlussheim, Germany) according to manufacturer's instructions [20]. Also a multicolor banding probe (MCB) sets based on microdissection derived region-specific libraries for chromosome 22 was applied as previously described [22]. A minimum of 10 metaphase spreads was analyzed, using a fluorescence microscope (AxioImager.Z1 mot, Carl Zeiss Ltd., Hertfordshir, UK) equipped with appropriate filter sets to discriminate between a maximum of five fluorochromes plus the counterstain DAPI (4',6- diamino-2-phenylindole). Image capture and processing were performed using an ISIS imaging system (MetaSystems, Altlussheim, Germany).

### Reverse transcriptase-polymerase chain reaction (RT-PCR) and for BCR/ABL1 fusion transcripts

Total RNA extracted from peripheral blood sample using the InviTrap RNA kit (Invitek, Berlin, Germany) according to the manufacturer’s recommendations. cDNA was prepared from 5 μg of total RNA with the Genequality BCR-ABL1 kit (AB Analitica, Padova, Italy) and BCR-ABL1 fusion transcript was performed according to the manufacturer’s instructions (AB Analitica, Padova, Italy).

### Consent

Written informed consent was obtained from the patient for publication of this Case Report. A copy of the written consent is available for review by the Editor-in-Chief of this journal.
